# The Duration of Intestinal Immunity After an Inactivated Poliovirus Vaccine Booster Dose in Children Immunized With Oral Vaccine: A Randomized Controlled Trial

**DOI:** 10.1093/infdis/jiw595

**Published:** 2016-12-21

**Authors:** Jacob John, Sidhartha Giri, Arun S Karthikeyan, Dipti Lata, Shalini Jeyapaul, Anand K Rajan, Nirmal Kumar, Pavithra Dhanapal, Jayalakshmi Venkatesan, Mohanraj Mani, Janardhanan Hanusha, Uma Raman, Prabhakar D Moses, Asha Abraham, Sunil Bahl, Ananda S Bandyopadhyay, Mohammad Ahmad, Nicholas C Grassly, Gagandeep Kang

**Affiliations:** 1 Department of Community Health, Vellore, Tamil Nadu; 2 Division of Gastrointestinal Sciences, Vellore, Tamil Nadu; 3 Department of Clinical Virology, Christian Medical College, Vellore, Tamil Nadu; 4 WHO Regional Office for South-East Asia, New Delhi, India; 5 Bill & Melinda Gates Foundation, Seattle, Washington; 6 WHO Country Office, New Delhi, India; 7 Department of Infectious Disease Epidemiology, Imperial College London, United Kingdom

**Keywords:** poliovirus, inactivated vaccine, oral vaccine, human challenge, mucosal immunity

## Abstract

**Background:**

In 2014, 2 studies showed that inactivated poliovirus vaccine (IPV) boosts intestinal immunity in children previously immunized with oral poliovirus vaccine (OPV). As a result, IPV was introduced in mass campaigns to help achieve polio eradication.

**Methods:**

We conducted an open-label, randomized, controlled trial to assess the duration of the boost in intestinal immunity following a dose of IPV given to OPV-immunized children. Nine hundred healthy children in Vellore, India, aged 1–4 years were randomized (1:1:1) to receive IPV at 5 months (arm A), at enrollment (arm B), or no vaccine (arm C). The primary outcome was poliovirus shedding in stool 7 days after bivalent OPV challenge at 11 months.

**Results:**

For children in arms A, B, and C, 284 (94.7%), 297 (99.0%), and 296 (98.7%), respectively, were eligible for primary per-protocol analysis. Poliovirus shedding 7 days after challenge was less prevalent in arms A and B compared with C (24.6%, 25.6%, and 36.4%, respectively; risk ratio 0.68 [95% confidence interval: 0.53–0.87] for A versus C, and 0.70 [0.55–0.90] for B versus C).

**Conclusions:**

Protection against poliovirus remained elevated 6 and 11 months after an IPV boost, although at a lower level than reported at 1 month.

**Clinical Trials Registration:**

CTRI/2014/09/004979.

The Global Polio Eradication Initiative (GPEI) has relied on oral poliovirus vaccines (OPVs) to eliminate wild-type (WT) polioviruses from most of the world. Only Afghanistan, Pakistan, and Nigeria remained endemic for WT poliovirus in 2016. OPV induces effective intestinal mucosal as well as humoral immunity against poliovirus infection. However, replication of the vaccine poliovirus is associated with a loss of attenuating mutations and, rarely, this reverted OPV may be transmitted and spread in the community as a circulating vaccine–derived poliovirus (cVDPV), causing outbreaks of poliomyelitis in settings of low immunization coverage [1, 2]. Therefore, the Global Polio Eradication Initiative (GPEI) has outlined a synchronized global withdrawal of OPV in its “Endgame Plan” (the Polio Eradication and Endgame Strategic Plan 2013–2018), starting with serotype 2 in April 2016 [3].

To mitigate the risks associated with OPV withdrawal, the World Health Organization (WHO) has recommended universal introduction of at least 1 dose of inactivated poliovirus vaccine (IPV) to infant immunization schedules worldwide [4]. A single dose of IPV administered at 14 weeks of age or later protects at least 50% of recipients against poliomyelitis and provides an immunity base for subsequent boosting with IPV if required [5]. However, IPV offers limited protection against poliovirus replication and shedding compared with OPV because it does not induce a mucosal immune response [6–8]. Therefore, it is possible for poliovirus to circulate in a population immunized solely with IPV without causing poliomyelitis, as was recently observed in Israel [9].

Although IPV does not induce intestinal mucosal immunity in naive children, it can boost immunoglobulin A (IgA) and gut-homing CD4^+^ T cells in children “mucosally primed” through past exposure to OPV or live polioviruses [10, 11]. In 2 recent clinical trials in India, a single dose of IPV given to OPV-immunized children substantially boosted protection against poliovirus shedding following a subsequent challenge with OPV [12, 13]. This boost was significantly greater than that offered by an additional dose of OPV. These results motivated the use of IPV in mass campaigns in Nigeria and Pakistan since early 2014 and Afghanistan since November 2014 to accelerate the elimination of both WT and vaccine-derived poliovirus transmission. Analysis of environmental and clinical surveillance data suggest that campaigns that used IPV alongside trivalent OPV have had a significant impact on poliovirus circulation in Nigeria but not in Pakistan, perhaps reflecting differences in the target age groups or in vaccine coverage [14]. Results from the clinical trials of IPV also led to IPV being included as a vaccine choice for travelers from endemic countries and now recommended for response against any outbreaks of type 2 cVDPV after withdrawal of serotype 2 OPV [15].

IPV is therefore now playing a critical role in the polio endgame through its distinct uses in routine infant immunization, and in mass campaigns to stop transmission of remaining WT polioviruses and newly emergent cVDPV. However, with severe global constraints on the supply of IPV, critical decisions need to be made about the allocation of IPV to campaigns and routine immunization [16]; in particular, the duration of the boost to intestinal immunity offered by IPV needs to be assessed to identify the optimal interval between IPV campaigns in a given area. Additionally, an estimate of the duration of protection will inform current recommendations under the International Health Regulations for immunization of travelers from countries exporting WT or cVDPV poliovirus, which currently require an IPV or OPV boost within 12 months before departure [17].

We therefore carried out a randomized controlled clinical trial to assess the duration of intestinal mucosal protection offered by a dose of IPV given to children previously immunized with OPV. Intestinal immunity was assessed by measuring poliovirus shedding after an OPV challenge dose given 6 or 11 months after IPV or after no vaccine (control). We compared protection at these time points with protection following challenge at 1 month after IPV, using data from our previously published study [13].

## METHODS

### Study Design and Participants

Children from Vellore, India, who had received at least 5 doses of trivalent OPV (tOPV) through routine and supplementary immunization were recruited to a parallel, open-label, randomized, controlled trial. Children were enrolled if they were between 12 and 59 months of age, were available for a year’s follow up and had no medical condition that precluded participation. Children were excluded if they had received IPV previously. Vaccination status was confirmed for each child based on their immunization card. At the time of the study, the recommended routine polio vaccine immunization schedule for enrolled children was tOPV given at birth, 6, 10, and 14 weeks, then a booster tOPV dose at 16–24 months of age. National immunization days, which occur twice annually, also used tOPV.

Written informed consent was obtained from the parent or legal guardian. The trial was conducted in accordance with the principles of good clinical practice and the ethical principles in the Declaration of Helsinki, with a protocol approved by the Institutional Review Board of the Christian Medical. Oversight of the study was provided by an independent data safety and monitoring board.

### Randomization and Masking

Children were randomized in a 1:1:1 ratio to receive a dose of IPV 5 months after enrollment (Arm A), at enrollment (Arm B), or no IPV (Arm C). A computer-generated block randomization with block sizes of 30 was generated by an independent statistician. The allocation codes in sequentially numbered opaque covers were opened at enrollment by study staff. All biological samples were masked with a unique laboratory ID such that laboratory staff performed blinded assessments.

### Procedures

A single intramuscular dose of IPV (Aventis Pasteur) was administered at 5 months (arm A) or at enrollment (arm B), containing 40, 8, and 32 D-antigen units of poliovirus serotypes 1, 2, and 3 respectively. A challenge dose of bivalent OPV (bOPV; Panacea Biotec), containing at least 10^6^ and 10^5.8^ median cell-culture infectious doses of Sabin serotypes 1 and 3 poliovirus was administered orally to all children at 11 months. We used bOPV for consistency with previous studies. Stool samples were collected just prior to and at 7, 14, and 21 days following the bOPV dose to assess poliovirus shedding. Serum-neutralizing antibody (NAb) titers were measured from blood samples collected 28 days after administration of IPV (arms A and B) or enrollment (arm C), and just prior to the bOPV dose.

Children were observed for 30 minutes after vaccine administration for allergic or adverse reactions. Study participants were eligible to receive 2 doses of tOPV during the national immunization days (NIDs) in January and February 2015. Scheduled booster doses of tOPV for children aged 16–24 months as part of the infant immunization schedule were withheld until study completion. Surveillance for serious adverse events (defined as adverse events that led to death, or were life threatening, or resulted in hospitalization) was performed through weekly home visits for 4 weeks following a vaccine dose, then subsequently through monthly telephone calls.

### Outcomes

The primary outcome of the study was the proportion of children shedding poliovirus 7 days after a challenge dose of serotypes 1 and 3 bOPV, administered 11 months after study enrollment. This challenge dose was given 1 month earlier than planned in the original study protocol, to ensure that it was given at the same time of year as in our previous study of poliovirus shedding 1 month after IPV [13]. This allowed for a comparison between these 2 studies that recruited (different) children from the same population without confounding seasonal effects on bOPV immunogenicity. Secondary outcomes included serum sample NAbs and, in a random subset of 150 infants, poliovirus shedding just prior to bOPV and at 14 and 21 days.

### Statistical Methods

The study was powered to detect a 40% relative reduction in shedding of poliovirus serotypes 1 or 3 in the IPV arms A and B from the expected level of 20% in the control arm C at 7 days after administration of the bOPV challenge dose. We calculated that for 80% power using the 2-sided Fisher exact test, we would need 281 children in each arm, which we inflated to 300 per arm to account for loss to follow up.

A per-protocol analysis was planned for all children who provided stool 7 days (up to 10 days) after bOPV challenge, a blood sample on the day of challenge, and who received IPV as planned. The proportion of children shedding serotypes 1 or 3 poliovirus and exact binomial 95% confidence intervals were calculated at each time point [18]. The Fisher exact test was used to compare the prevalence of shedding 7 days after challenge between study arms for the primary analysis. Risk ratios for shedding were calculated together with Wald-method 95% confidence intervals [19]. The mean and standard error of the virus copy number were calculated on a log scale, and differences among the study arms were assessed using the Kruskal–Wallis nonparametric test. Correlation in shedding of poliovirus serotypes 1 and 3 was determined based on the φ-coefficient.

The median titer of serum sample NAbs to each poliovirus serotype was calculated using the Spearman–Karber method [20]. Geometric mean antibody titers (GMTs) were calculated by assigning a value of 1/6 and 1/1448 for the censored values below and above the limits of the dilution series. Antibody titers were compared between study arms and by poliovirus shedding status using the nonparametric Wilcoxon rank-sum test. Potential associations between the baseline characteristics of enrolled children and the decline in antibody titer, measured as the difference in the natural log of the reciprocal antibody titer 28 days after IPV or no vaccine and at the time of bOPV challenge, were assessed using univariable and multivariable linear regression.

### Laboratory Methods

Serum samples were tested for poliovirus-specific NAbs to types 1, 2 and 3 using microneutralization as recommended by the WHO [21]. Samples were tested in 2-fold serial dilutions from 1/8 to 1/1024. Shedding of poliovirus in stool samples was assessed using quantitative real-time polymerase chain reaction (PCR) [22]. Further details are given in the Supplementary Methods.

## RESULTS

### Study Procedures

**Figure 1.**
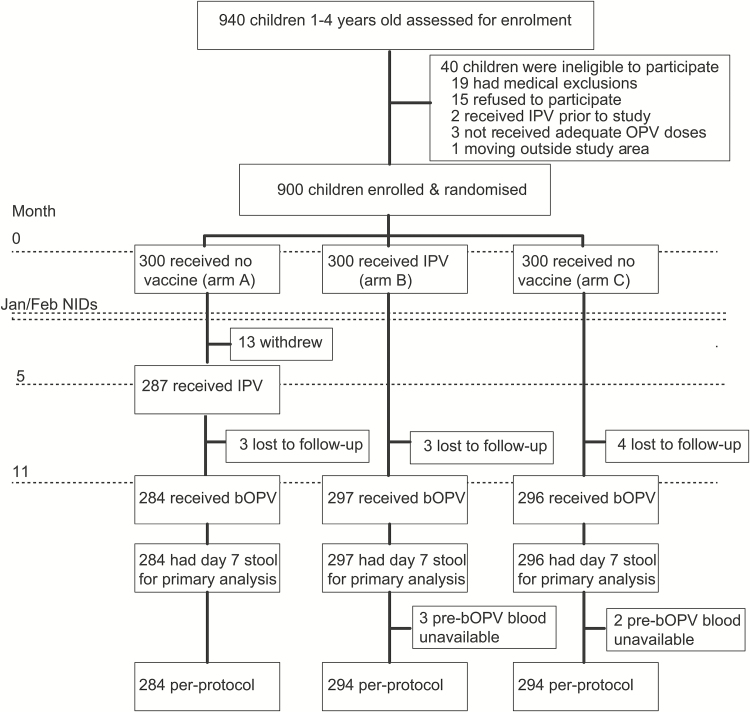
Trial profile. Abbreviations: bOPV, serotypes 1 and 3 bivalent oral poliovirus vaccine; IPV, inactivated poliovirus vaccine; NID, National Immunization Day (which took place on 19 January and 23 February 2015).

Nine hundred children were recruited between 4 November and 17 December 2014, and randomly assigned to one of the 3 study groups (Table 1). All study procedures were completed by 10 December 2015. Eight hundred seventy-seven children who had received their primary interventions as planned received the challenge dose of bOPV at 11 months (Figure 1). All 877 children provided a stool sample 7 days later and were eligible for intention-to-treat analysis. Of these, 872 (99.4%) also provided a blood sample on the day of challenge and were eligible for the primary per-protocol analysis. The median time between vaccination with IPV and bOPV challenge was 189 days (5th and 95th percentiles were 184 and 195 days, respectively) in arm A and 330 days in arm B (5th and 95th percentiles, 323 and 339 days, respectively); the median time between enrollment and bOPV challenge in arm C was 330 days (5th and 95th percentiles, 322 and 338 days, respectively). Eight hundred ninety-four (99.3%) children received at least 1 of the 2 tOPV doses administered during NIDs, while 867 (96.3%) received both doses. These occurred prior to the IPV dose in arm A (Figure 1).

**Table 1.** Baseline Characteristics of Infants Enrolled in the Study

Characteristics	Arm A (IPV 6 mo. before challenge)	Arm B (IPV 11 mo. before challenge)	Arm C (no IPV)
Demography and anthropometry:			
Age (y)	2.76 (0.06)	2.62 (0.06)	2.8 (0.06)
Female	173 (57.7)	176 (58.7)	144 (48)
Weight (kg)	11.38 (0.14)	11.33 (0.14)	11.59 (0.13)
Height (cm)	86.71 (0.57)	86.06 (0.56)	87.25 (0.54)
Mother’s education (5th grade or lower)	92 (30.7)	76 (25.3)	105 (35)
House roof concrete (vs thatch or similar)	186 (62)	180 (60)	191 (63.7)
Vaccination history:			
Number of trivalent oral poliovirus vaccine doses received	8.31 (0.12)	7.93 (0.11)	8.26 (0.11)
Time since last oral poliovirus vaccine (mo.)	8.12 (0.15)	8.03 (0.13)	8.3 (0.09)

Data are mean (standard error) or no. (%).

Abbreviation: IPV, inactivated poliovirus vaccine.

### Poliovirus Shedding

**Figure 2.**
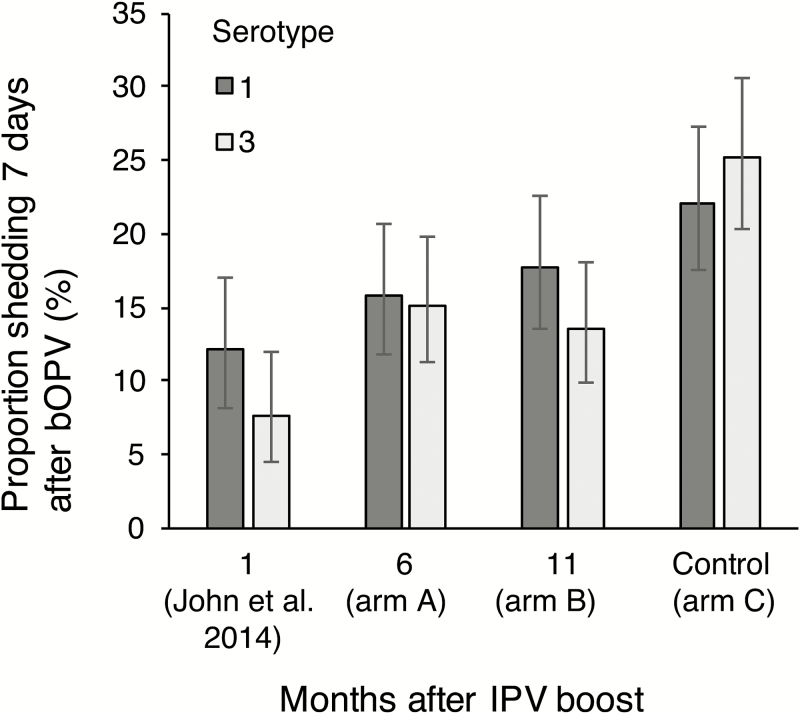
Prevalence of poliovirus shedding on day 7 after bOPV challenge according to the time since receipt of an IPV booster dose. The data for shedding challenge virus at 1 month after an IPV boost were collected 2 years prior to the current study and have previously been reported [13]. The fit of a simple linear model to these data imply a loss of protection against shedding at a rate of approximately 0.6% per month in absolute terms for either serotype. Abbreviations: bOPV, serotypes 1 and 3 bivalent oral poliovirus vaccine; IPV, inactivated poliovirus vaccine.

Seven days after challenge, the proportion of children shedding serotypes 1 and/or 3 poliovirus was significantly lower in arms A and B compared with control arm C (risk ratio [RR] 0.68 [95% confidence interval {CI}, 0.53–0.87]; Fisher *P* = .003 and 0.70 [0.55–0.90], Fisher *P* = .006 for arm A vs C and B vs C, respectively; Table 2). The reduction in shedding was more marked for serotype 3 (RR 0.60 [0.43–0.84], *P* = .004; and RR 0.54 [0.38–0.77], *P* = .001, respectively) than for serotype 1 (RR 0.72 [0.51–1.01], *P* = .057; and RR 0.80 [0.58–1.11], *P* = .215, respectively). Very similar results were obtained in the intention-to-treat analysis (Supplementary Table 1). Poliovirus shedding as a function of time since receipt of IPV is shown in Figure 2, which includes data from our previously published study of bOPV challenge at 1 month (conducted in the same location) [13]. Both studies administered bOPV at a similar time of the year (median date 14 October 2015 [interquartile range {IQR}: 5 October–21 Oct 2015] for the current study compared with 27 September 2013 [IQR: 20 September –4 October 2013] for the previous study).

**Table 2.** Poliovirus Shedding 7 Days After bOPV Challenge

	Arm A (n = 284) (IPV 6 mo. before challenge)	Arm B (n = 294) (IPV 11 mo. before challenge)	Arm C (n = 294) (no IPV)
Number shedding (%):			
Serotype 1 or 3	70 (24.6)	75 (25.6)	107 (36.4)
Serotype 1	45 (15.8)	52 (17.7)	65 (22.1)
Serotype 3	43 (15.1)	40 (13.6)	74 (25.2)
Mean log_e_ viral copy number among those shedding (SE):			
Serotype 1	6.03 (0.35)	5.83 (0.45)	5.53 (0.34)
Serotype 3	8.30 (0.36)	7.94 (0.46)	8.16 (0.36)

Abbreviations: bOPV, bivalent oral polio vaccine; IPV, inactivated poliovirus vaccine; SE, standard error.

The estimated mean virus copy number among children shedding poliovirus did not differ by study arm (Kruskal–Wallis test *P* = .584 and 0.949 for serotypes 1 and 3 respectively; Table 2). Seven days after challenge, shedding of serotype 1 and of serotype 3 poliovirus were correlated (φ coefficient = 0.29; *P* < .001).

In the subset of 150 children tested for poliovirus shedding at additional time points, 1 (0.7%) child in arm C was shedding (serotype 1) on the day of challenge. In the same subset, the prevalence of serotype 1 or 3 poliovirus shedding declined on day 14 and 21, compared with day 7 (Supplementary Figure 1). The prevalence of poliovirus shedding on days 14 and 21 did not show significant differences by study arm (Fisher *P* = .844 and 1.000, respectively).

### Serum-Neutralizing Antibodies

**Figure 3.**
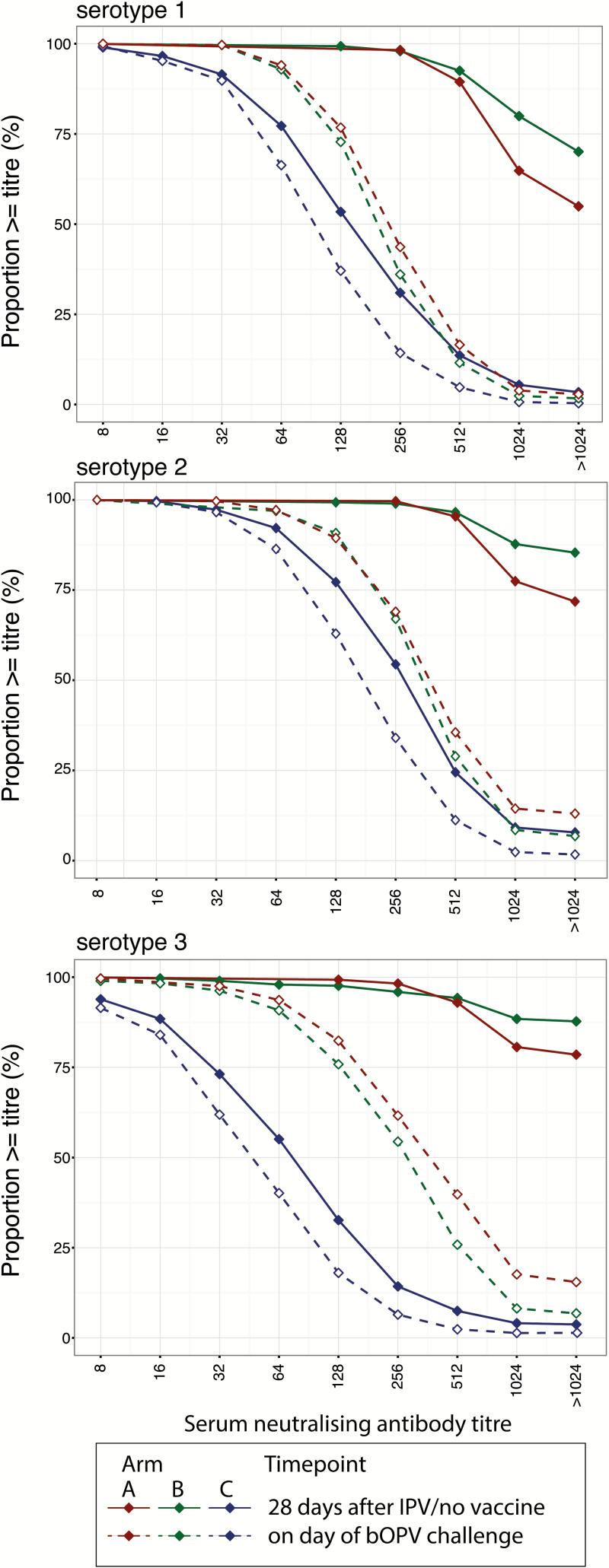
Distribution of serum-neutralizing antibody titers. The reverse cumulative distribution of antibody titers is shown for each study arm 28 days after IPV (arms A and B) or no vaccine (arm C) (solid lines) and at the time of bOPV challenge (dashed lines). Abbreviations: bOPV, serotypes 1 and 3 bivalent oral poliovirus vaccine; IPV, inactivated poliovirus vaccine.

Serum NAb titers were significantly higher 28 days after IPV in arms A and B compared to 28 days after enrollment in control arm C (Wilcoxon rank sum [WRS] *P* values all <.001; Table 3, Figure 3). The NAb titers waned significantly by the time of bOPV challenge in all 3 arms (Wilcoxon signed rank test *P* values all <.001 for each serotype in all 3 arms). The drop in NAb titer among children receiving IPV was greater in children with a higher starting titer, greater in arm B (after 10 months) compared with A (after 5 months) and, for serotype 2 only, appeared to be more rapid in older children (Supplementary Table 2). Nonetheless, NAb titers remained significantly higher in arms A and B compared with control arm C at the time of bOPV challenge (WRS test *P* values all <.001; Table 3). For both serotypes 1 and 3, serum NAb titers at the time of bOPV challenge tended to be lower in children who subsequently shed this serotype of poliovirus than in nonshedders (Table 3; Supplementary Figure 2). Among those children with a reciprocal titter ≥256, 11.4% and 13.3% shed serotypes 1 and 3 poliovirus, respectively, 7 days after bOPV challenge, compared with 21.8% and 21.2% of those with titers below this threshold.

**Table 3.** Serum-Neutralizing Antibody Titers by Study Arm and Time Point

	Arm A (IPV 6 mo. before challenge)	Arm B (IPV 11 mo. before challenge)	Arm C (no IPV)
28 days after IPV (arms A and B) or enrollment (arm C):			
Serotype 1	891.0	1058.3	103.9
Serotype 2	1085.7	1220.5	191.8
Serotype 3	1100.4	1149.3	53.5
At time of bOPV challenge:			
All children			
Serotype 1	164.2	143.2	67.6
Serotype 2	277.8	248.3	122.5
Serotype 3	255.7	184.3	34.5
At time of bOPV challenge—according to homologous poliovirus shedding on day 7:			
Serotype 1 shedders	129.0	102.0	61.0
Serotype 1 nonshedders	171.8	154.0	69.7
*P* value	.025	.001	.269
Serotype 3 shedders	194.6	136.0	26.9
Serotype 3 nonshedders	268.4	193.3	37.5
*P* value	.104	.137	.022

Data are geometric mean of the (reciprocal) titer (GMT).

Abbreviations: bOPV, bivalent oral polio vaccine; IPV, inactivated poliovirus vaccine.

### Safety

Forty-one serious adverse events (11 in arm A, 17 in arm B, and 13 in arm C), including 2 deaths in arm A, were reported during the trial. All reported adverse events were classified as unrelated. The 2 deaths were from leukemia and from viral hemorrhagic fever.

## DISCUSSION

The boost to intestinal immunity against poliovirus that results from administration of IPV to children vaccinated with OPV is sustained at 6 and 11 months. Nonetheless, the degree of mucosal protection was less than that observed 1 month after an IPV boost [13], and appears to wane quite rapidly (Figure 2). Linear extrapolation of the observed trends suggests that protection wanes to levels observed in the control arm about 2 years after administration of IPV. These results are consistent with the observation of diminished intestinal immunity to poliovirus about a year after vaccination with OPV [23]. Protection against poliovirus shedding was apparent for stool samples taken 7 days after OPV challenge, but not at 14 and 21 days, perhaps as a result of the smaller number of children assessed at these time points, the lower prevalence of shedding, and limited statistical power.

Waning protection against poliovirus shedding was mirrored by trends observed for serum-neutralizing antibodies. Antibody titers were substantially higher among children in the IPV study arms compared with the control arm 28 days after vaccination (reciprocal GMTs all >1000 for all 3 serotypes in the IPV arms compared with 53.5–191.8 in the control arm). These levels had dropped substantially by the time of OPV challenge 5 or 10 months later, but remained elevated compared with the control arm (143.2–277.8 compared with 34.5–122.5, depending on serotype and study arm). Antibody titers were significantly lower among children who shed poliovirus after challenge compared with those who did not, consistent with studies among children immunized with OPV [24, 25]. This suggests that among children who have been “mucosally primed” through exposure to OPV or live poliovirus, serum-neutralizing antibodies may be a useful nonmechanistic, relative correlate of protection (CoP) against intestinal poliovirus infection (as well as a mechanistic, absolute CoP against poliomyelitis) [26].

In this study, the majority of children received 1 or 2 doses of trivalent OPV through NIDs that took place in January and February 2015, and in arm B these occurred between the receipt of IPV and OPV challenge. Despite the potential boost to intestinal immunity offered by the trivalent OPV dose in all three arms, we still observed a significant difference between children in arm B (and A) compared with those in the control arm C. This indicates that the boost offered by IPV is greater than that offered by further doses of OPV, in agreement with our earlier observations [12, 13]. It is possible that in the absence of intervening NIDs, the difference between study arms would have been greater. However, we decided not to restrict access to OPV through NIDs among children enrolled in the study, because this better reflects programmatic use of IPV in mass campaigns where intervening use of OPV is common. Moreover, children living in communities using OPV in their routine schedules may be exposed to vaccine poliovirus following secondary spread from vaccinated siblings or other contacts. Secondary spread in our study appeared to be limited, because only 1 (0.7%) child was shedding poliovirus on the day of OPV challenge.

Our study had a number of limitations. We used PCR to assess poliovirus shedding in stool rather than growth in cell culture, which would confirm the presence of infectious virus. However, in previous work, PCR and culture growth results were strongly correlated, indicating that PCR is a suitable assay to assess poliovirus shedding in challenge studies.[13] It is also possible that the duration of intestinal immunity to poliovirus differs according to study population. We examined potential correlates with the rate of decline in serum-neutralizing antibodies, and did not identify any significant demographic or socioeconomic correlates. Moreover, our results are consistent with observations following OPV immunization in northern India, suggesting that they are relevant at least nationally and most likely in other low-income settings [23].

Our findings have implications for the GPEI endgame strategy. First, they indicate that further doses of IPV, 1–2 years after an initial boost of OPV, may be required to sustain a high level of intestinal immunity to poliovirus. Annual campaigns with IPV in areas with persistent WT or vaccine-derived poliovirus transmission would achieve this, while also offering additional chances to vaccinate children missed in earlier campaigns and reaching children in the birth cohort not previously targeted with IPV. The optimal frequency of campaigns will depend on local considerations, concerning campaign coverage, birth rates, and patterns of travel and migration. High coverage during mass campaigns is essential, not only to ensure children receive IPV but because in the absence of good immunization coverage, fewer children will have been mucosally primed through exposure to OPV.

Second, the findings are important from the perspective of outbreak response strategy following the global withdrawal of OPV that began with serotype 2 in April 2016. The current recommendations include the use of IPV as an adjunct to monovalent OPV in mass campaigns that respond to any circulating serotype 2 poliovirus [15].Thus, understanding the dynamics of the IPV boost to intestinal immunity and its duration in such situations helps inform the optimum use of IPV to sustain the interruption of transmission and minimize the risk of reintroduced OPV seeding the circulation of vaccine-derived viruses or generating vaccine-associated cases. This role of IPV will change as the polio endgame progresses because the cohort of children born after the global withdrawal of serotype 2 OPV will not have been mucosally primed against this serotype. Therefore, the impact of campaigns that use IPV will change as a function of time since OPV withdrawal, depending on the balance between the growth of this cohort and the magnitude of the IPV boost among older children with waning mucosal protection.

Third, the finding of waning immunity supports the requirement for re-vaccination with IPV if 12 months have passed since the last dose, as recommended by the International Health Regulations for travelers from countries exporting WT or vaccine-derived polioviruses. Finally, the data suggest that population surveys of serum-neutralizing antibodies to poliovirus may be useful in high-risk areas using both OPV and IPV, not only as an indicator of protection against poliomyelitis but also as an indirect measure of intestinal immunity against infection.

In conclusion, it is clear that IPV is playing an increasingly important role in the polio endgame as the world transitions away from the use of OPV. Every effort needs to be made to ensure supply of this vaccine is available to meet this expanding role.

## Supplementary Data

Supplementary materials are available at *The Journal of Infectious Diseases* online. Consisting of data provided by the authors to benefit the reader, the posted materials are not copyedited and are the sole responsibility of the authors, so questions or comments should be addressed to the corresponding author.

## Supplementary Material

Supplementary Figure 1

Supplementary Figure 2

Supplementary Table 1

Supplementary Table 2

Supplementary Methods

## Notes

***Disclaimer.*** All authors had full access to data and shared responsibility for the decision to submit for publication.

***Financial support.*** This work was supported by the Bill & Melinda Gates Foundation (grant no. OPP1108936).

***Potential conflicts of interest.*** A. S. B. is an employee of the study funder, and was involved in study design and data interpretation. All other authors report no potential conflicts. All authors have submitted the ICMJE Form for Disclosure of Potential Conflicts of Interest. Conflicts that the editors consider relevant to the content of the manuscript have been disclosed.
