# Supplementary material for: The Duration of Intestinal Immunity After an Inactivated Poliovirus Vaccine Booster Dose in Children Immunized With Oral Vaccine: A Randomized Controlled Trial
Source: J Infect Dis. 2016 Dec 21;215(4):529–36. doi: 10.1093/infdis/jiw595 (PMC5388294; doi:10.1093/infdis/jiw595)
Supplement: Supplementary Figure 1 [file jiw595_suppl_supplementary_figure_1.docx]

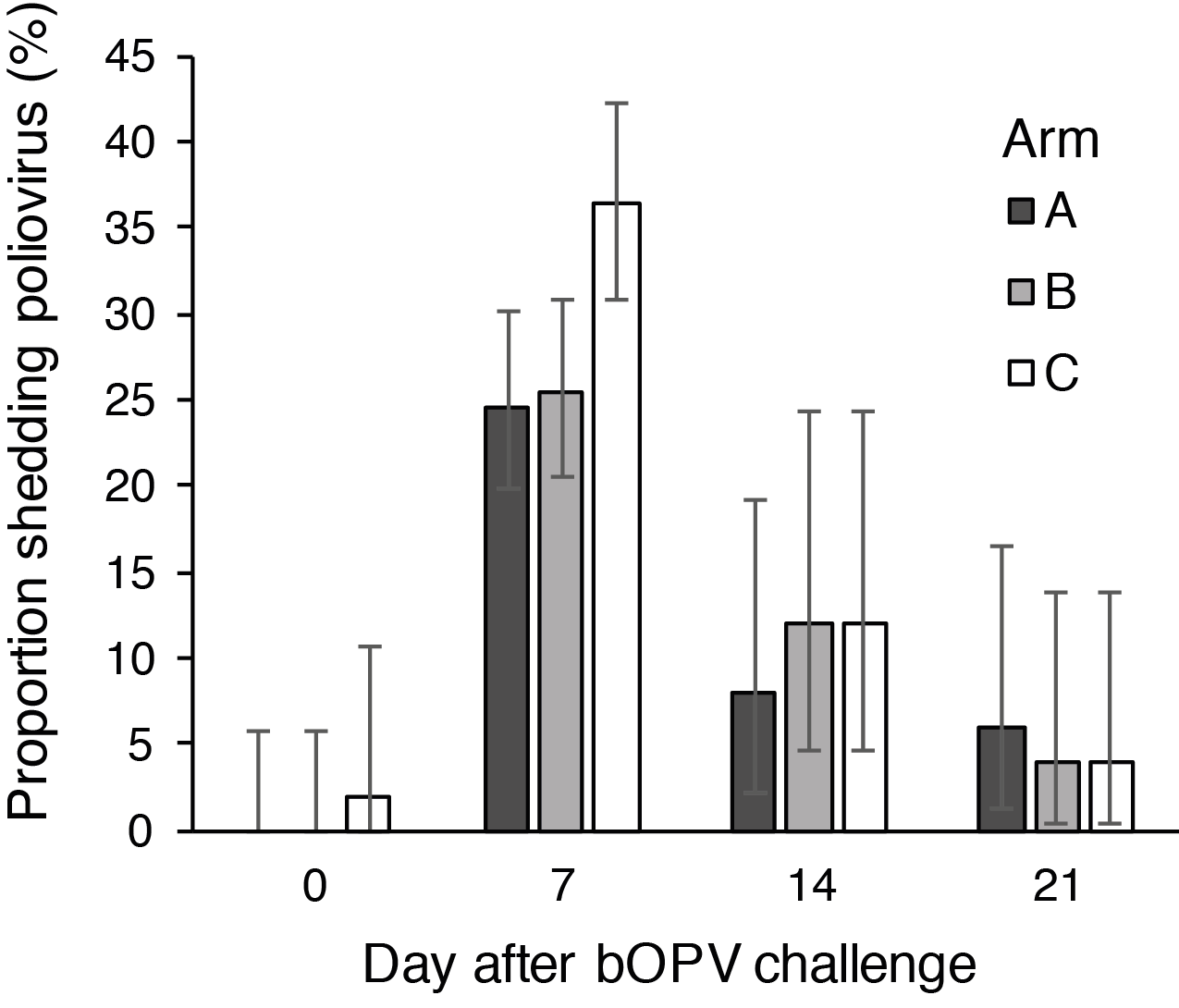


**Supplementary Figure 1** Prevalence of serotype 1 or 3 poliovirus shedding by study arm shown as a function of time since challenge. Data for stools collected just before bivalent oral poliovirus vaccine administration (day 0) and on days 14 and 21 are based on testing of a subset of 150 infants.
