# Supplementary material for: The Duration of Intestinal Immunity After an Inactivated Poliovirus Vaccine Booster Dose in Children Immunized With Oral Vaccine: A Randomized Controlled Trial
Source: J Infect Dis. 2016 Dec 21;215(4):529–36. doi: 10.1093/infdis/jiw595 (PMC5388294; doi:10.1093/infdis/jiw595)
Supplement: Supplementary Figure 2 [file jiw595_suppl_supplementary_figure_2.docx]

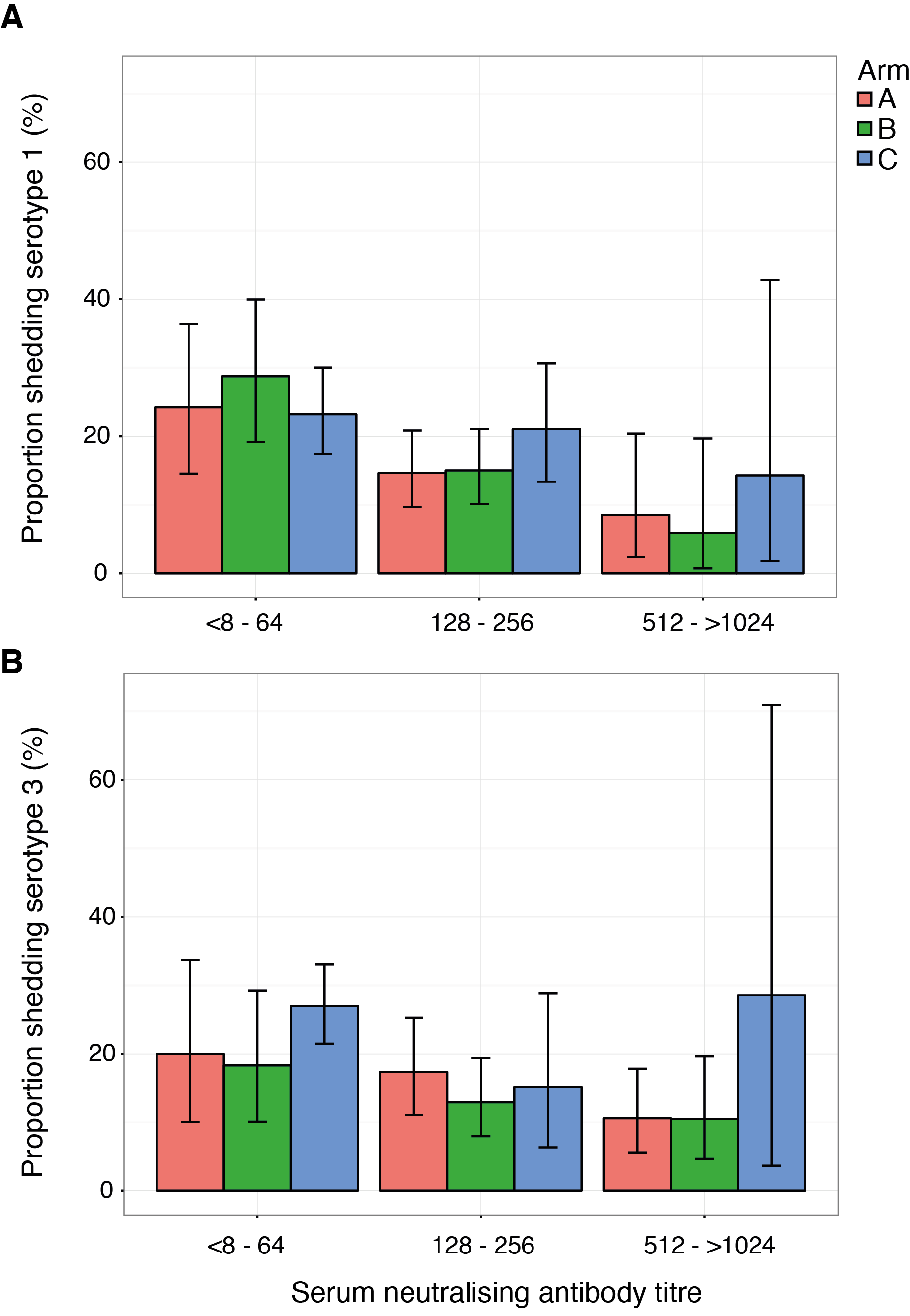


**Supplementary Figure 2** Prevalence of (A) serotype 1 and (B) serotype 3 poliovirus shedding by study arm shown as a function of serum neutralising antibody titre at the time of challenge with bivalent oral poliovirus vaccine.
