# Supplementary material for: The Duration of Intestinal Immunity After an Inactivated Poliovirus Vaccine Booster Dose in Children Immunized With Oral Vaccine: A Randomized Controlled Trial
Source: J Infect Dis. 2016 Dec 21;215(4):529–36. doi: 10.1093/infdis/jiw595 (PMC5388294; doi:10.1093/infdis/jiw595)
Supplement: Supplementary Table 1 [file jiw595_suppl_supplementary_table_1.docx]

**Supplementary Table 1 Poliovirus shedding 7 days after bOPV challenge (ITT analysis)**

|  | **Arm A (n=284)** |  | **Arm B (n=297)** | **Arm C (n=296)** |
| --- | --- | --- | --- | --- |
| *Number shedding (%)* | | | | |
| Serotype 1 or 3 | 70 (24.6) |  | 77 (25.9) | 108 (36.5) |
| Serotype 1 | 45 (15.8) |  | 53 (17.8) | 66 (22.3) |
| Serotype 3 | 43 (15.1) |  | 41 (13.8) | 74 (25.0) |
| *Mean log_e_ viral copy number among those shedding (SE)* | | | | |
| Serotype 1 | 6.03 (0.36) |  | 5.81 (0.44) | 5.51 (0.33) |
| Serotype 3 | 8.30 (0.36) |  | 7.88 (0.46) | 8.16 (0.36) |
