# Supplementary material for: The Duration of Intestinal Immunity After an Inactivated Poliovirus Vaccine Booster Dose in Children Immunized With Oral Vaccine: A Randomized Controlled Trial
Source: J Infect Dis. 2016 Dec 21;215(4):529–36. doi: 10.1093/infdis/jiw595 (PMC5388294; doi:10.1093/infdis/jiw595)
Supplement: Supplementary Table 2 [file jiw595_suppl_supplementary_table_2.docx]

**Supplementary Table 2 Univariable and multivariable linear regression analysis of the difference in the natural log of the reciprocal neutralising antibody titre between the first and second blood draws in arms A and B**

|  | **Serotype 1** | | | |  | **Serotype 2** | | | |  | **Serotype 3** | | | |  |
| --- | --- | --- | --- | --- | --- | --- | --- | --- | --- | --- | --- | --- | --- | --- | --- |
|  | **Univariable** | | **Multivariable** | | | **Univariable** | | **Multivariable** | | | **Univariable** | | **Multivariable** | | |
| **Variable** | **coefficient** | **p-value** | **coefficient** | **p-value** | | **coefficient** | **p-value** | **coefficient** | **p-value** | | **coefficient** | **p-value** | **coefficient** | **p-value** | |
| arm A vs. B | 0.446 | <0.001 | 0.338 | <0.001 | | 0.331 | 0.001 | 0.313 | 0.001 | | 0.535 | <0.001 | 0.527 | <0.001 | |
| age (years) | -0.034 | 0.443 | - | - | | -0.144 | 0.002 | -0.144 | 0.001 | | -0.048 | 0.375 | - | - | |
| baseline titre | -0.459 | <0.001 | -0.433 | <0.001 | | -0.282 | <0.001 | -0.242 | <0.001 | | -0.138 | 0.021 | -0.129 | 0.0278 | |
| male sex | 0.043 | 0.664 | - | - | | -0.012 | 0.905 | - | - | | 0.248 | 0.037 | - | - | |
| height (cm) | -0.003 | 0.512 | - | - | | -0.014 | 0.006 | - | - | | 0 | 0.955 | - | - | |
| weight (kg) | 0.000 | 0.523 | - | - | | 0 | 0.019 | - | - | | 0 | 0.883 | - | - | |
| mother’s education | 0.073 | 0.499 | - | - | | -0.044 | 0.693 | - | - | | -0.021 | 0.871 | - | - | |
| house type | -0.008 | 0.936 | - | - | | 0.016 | 0.874 | - | - | | 0.049 | 0.686 | - | - | |
| number of OPV doses received | -0.008 | 0.757 | - | - | | -0.083 | 0.001 | - | - | | -0.017 | 0.576 | - | - | |
| time since last OPV dose | -0.001 | 0.417 | - | - | | -0.001 | 0.379 | - | - | | -0.001 | 0.064 | - | - | |
