# Supplementary material for: The Duration of Intestinal Immunity After an Inactivated Poliovirus Vaccine Booster Dose in Children Immunized With Oral Vaccine: A Randomized Controlled Trial
Source: J Infect Dis. 2016 Dec 21;215(4):529–36. doi: 10.1093/infdis/jiw595 (PMC5388294; doi:10.1093/infdis/jiw595)
Supplement: Supplementary Methods [file jiw595_suppl_supplementary_methods.docx]

**Supplementary Methods**

*Laboratory methods*

Blood samples were centrifuged at 2000 rpm for 15 minutes to obtain serum, which was frozen within 4 hours and kept at -20°C until testing. Serum was tested for poliovirus-specific neutralizing antibodies to types 1, 2 and 3 using a microneutralisation assay as recommended by the WHO with modifications.^1^ Samples were tested in two-fold serial dilutions from 1/8 to 1/1024.

Stool samples were aliquoted and frozen at -70°C within 8 hours of collection prior to testing. For analysis of shedding, a multiplex one-step quantitative reverse transcription real-time PCR (qRT-PCR) for Sabin poliovirus 1, 2, 3, and MS2 bacteriophage (used as extraction control) was carried out, using RNA extracted using Vx reagents on a Qiaxtractor.^2,3^ Before extraction, each sample was spiked with MS2 calibrated to yield a Ct value at 25-26 cycles. Reverse transcription and DNA amplification was carried out in an ABI thermal cycler with detection using Taqman probe hybridization. Plasmids constructed by ligation of a poliovirus 1 and a poliovirus 3 region of the VP1 PCR fragment in TOPO-TA 2.1 vector propagated in *Escherichia coli* DH5α cells were used as plasmid DNA standards for calibration of assay interpretation. Threshold cycle (Ct) values of <37 for Sabin 1 and <39 for Sabin 3 were considered positive for poliovirus. All stool samples were checked to be positive for MS2 to rule out inhibition.

*References*

1. World Health Organisation. Manual of laboratory methods for testing of vaccines used in the WHO Expanded Programme on Immunization. WHO/VSQ/97.04. 1997.

2. Taniuchi M, Begum S, Uddin MJ, et al. Kinetics of Poliovirus Shedding following Oral Vaccination as Measured by Quantitative Reverse Transcription-PCR versus Culture. *J Clin Microbiol* 2015; **53**(1): 206-11.

3. Kilpatrick DR, Yang C-F, Ching K, et al. Rapid group-, serotype-, and vaccine strain-specific identification of poliovirus isolates by real-time reverse transcription-PCR using degenerate primers and probes containing deoxyinosine residues. *J Clin Microbiol* 2009; **47**(6): 1939-41.
